# Primary Retroperitoneal Mucinous Cystadenocarcinoma in Males: A Case Report

**DOI:** 10.7759/cureus.95765

**Published:** 2025-10-30

**Authors:** Anansh Badhwar, Mohim Thakur, Saumya Chopra, Lokesh Rana, Manupriya Sharma

**Affiliations:** 1 Department of General Surgery, All India Institute of Medical Sciences Bilaspur, Bilaspur, IND; 2 Department of General Surgery, All India Institute of Medical Sciences Nagpur, Nagpur, IND; 3 Department of Diagnostic Radiology, All India Institute of Medical Sciences Bilaspur, Bilaspur, IND; 4 Department of Pathology, All India Institute of Medical Sciences Bilaspur, Bilaspur, IND

**Keywords:** cystic tumor, mucinous tumor, pmrc, primary retroperitoneal cystic tumor, retroperitoneal tumor

## Abstract

Primary retroperitoneal mucinous cystadenocarcinoma (PRMC) is a rare malignant neoplasm, predominantly reported in women, with occurrence in men being extremely uncommon. A handful of male cases have been described in the literature. The deep retroperitoneal location and nonspecific symptoms often delay diagnosis and complicate management. We report the case of a 69-year-old male presenting with a four-month history of abdominal discomfort and significant unintentional weight loss. Imaging with CT and MRI demonstrated a large, multiloculated cystic retroperitoneal mass causing displacement of the right kidney, ureter, and inferior vena cava. The patient underwent an exploratory laparotomy with complete excision, along with right hemicolectomy and ileocolic anastomosis. Histopathology confirmed moderately differentiated mucinous cystadenocarcinoma. Immunohistochemistry was suggestive of intestinal-type differentiation. Postoperative recovery was uneventful, and the patient was started on adjuvant chemotherapy. PRMC in males is rare and potentially aggressive. Complete surgical resection with negative margins remains the cornerstone of management, while adjuvant chemotherapy is considered in high-risk cases.

## Introduction

Retroperitoneal tumors are rare and account for less than 0.2% of all neoplasms. It includes a heterogeneous group of lesions originating in the retroperitoneal space. The majority are malignant and commonly are mesenchymal in origin. They can be primary or secondary, i.e., primary lesions are those that do not originate from organs such as the kidneys, pancreas, adrenal glands, or bowel loops. Retroperitoneal mass presents with nonspecific symptoms influenced by its location and relation to the nearby structures. Key imaging modalities for further evaluation include a CT scan or an MRI [1]. The treatment of retroperitoneal masses is tricky due to their anatomical location, dimensions, vascular involvement, and infiltration of adjacent organs [2].

Primary retroperitoneal mucinous cystadenocarcinoma (PRMC) is an uncommon neoplasm, with the majority of reported cases occurring in women. Its occurrence in men is extremely rare [3]. Due to its deep anatomical relation and non-specific symptoms, diagnosing PRMC is often challenging. Our case highlights the diagnostic journey and management of a 69-year-old male with a large retroperitoneal mucinous cystadenocarcinoma.

## Case presentation

A 69-year-old male presented to the outpatient clinic of our department with the complaint of abdominal discomfort for the last four months and weight loss of around 10 kg in the last two months. The pain was described as dull, poorly localized, constant, and non-radiating, with no aggravating or relieving factors. Over the past two months, the patient reported a history of significant unintentional weight loss (more than 10 kg), associated with reduced appetite and generalized fatigue. There was no history of any other gastrointestinal symptoms. The patient had a past history of diabetes mellitus and hypertension, and was a chronic smoker, and he had a history of alcohol intake.

Abdominal examination revealed a visible lump in the right lumbar region. On palpation, the lump was not tender; it measured approximately 15 × 12 cm and had ill-defined margins, though getting above and below the lump was possible; its surface was smooth, and there was no movement with respiration. Chest examination was normal.

**Figure 1:**
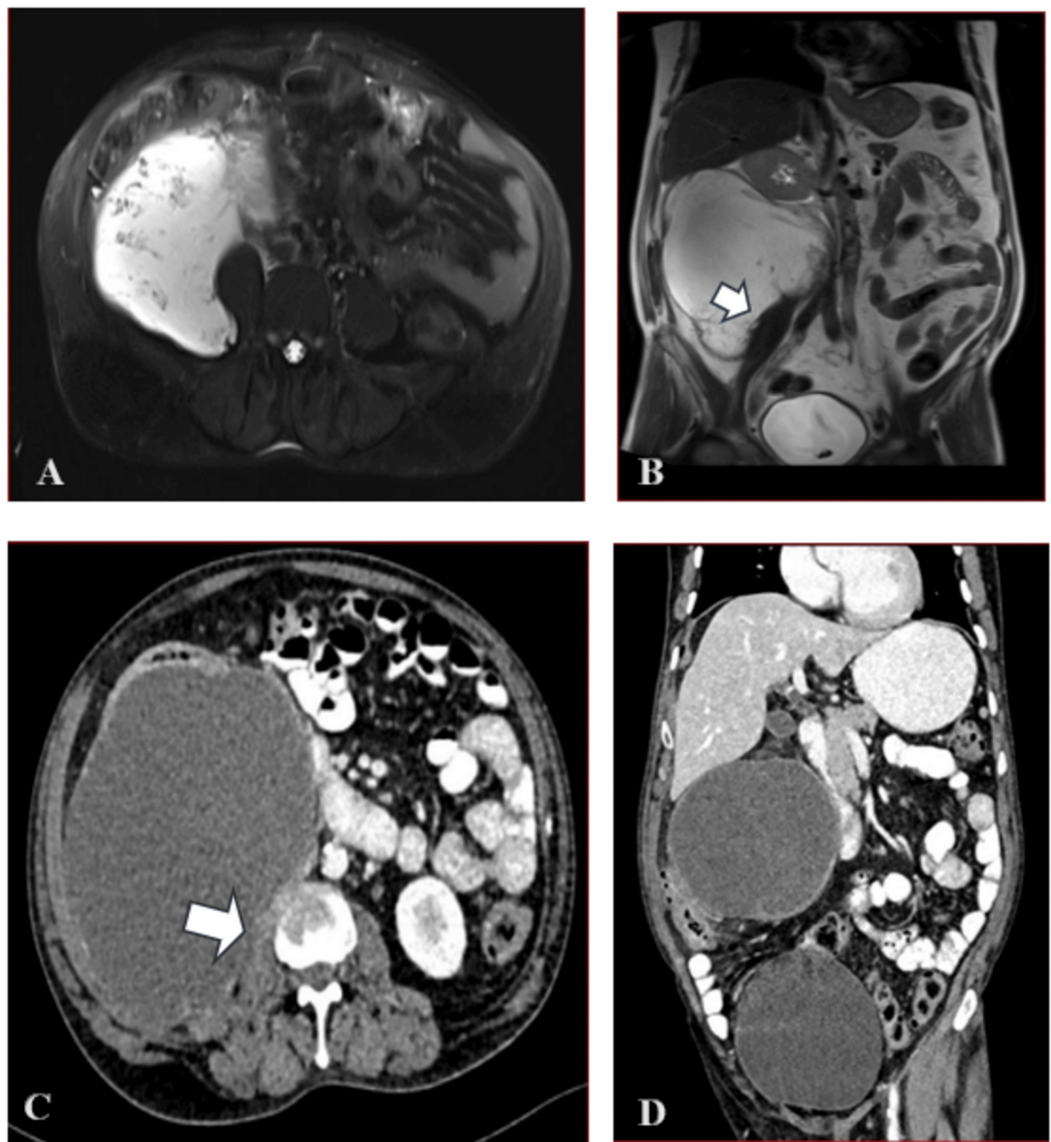
MRI and CT Images A) T2-weighted axial MRI image of the abdomen showing a hyperintense cystic lesion. B) T2-weighted coronal MRI image of the abdomen showing a large, well-circumscribed cystic lesion displacing the left kidney upwards (homogenous hyperintense). C) Contrast-enhanced CT of the abdomen showing a 16 cm × 15 cm homogenous cystic mass in the retroperitoneal space with displacement of the ascending colon with a solid component in the cyst wall  (white arrow). D) CT abdomen showing a well-circumscribed, homogenous hypodense lesion in the right hepatic and pelvic cavity.

Blood panel showed raised tumor markers (CEA = 442 ng/mL and CA 19-9 = 905 U/mL). Abdomen and chest CT revealed a 16 × 15 × 18 cm well-defined, multiloculated abdomino-pelvic hypodense retroperitoneal cystic lesion on the right side. MRI of abdomen and pelvis revealed similar findings with the differential diagnosis as cystic retroperitoneal neoplasm (Figures 1A-1B). Peripheral rim calcification and internal septal calcification were noted in the inferior aspect (Figures 1C-1D). No fat density was seen. Significant mass effect was noted in the form of anteromedial displacement of the right kidney with loss of fat planes. Superiorly, the cyst was extending up to the inferior surface of rt lobe of the liver. No obvious communication with the bowel was seen. Medially, the right ureter and inferior vena cava (IVC) are compressed with maintained fat planes. The right kidney was medially displaced and horizontally oriented, with the lesion closely abutting its lateral surface. Fat plans were maintained with the anterior abdominal wall. The lesion was abutting the external iliac artery and vein (no vascular encasement was seen). 

**Figure 2:**
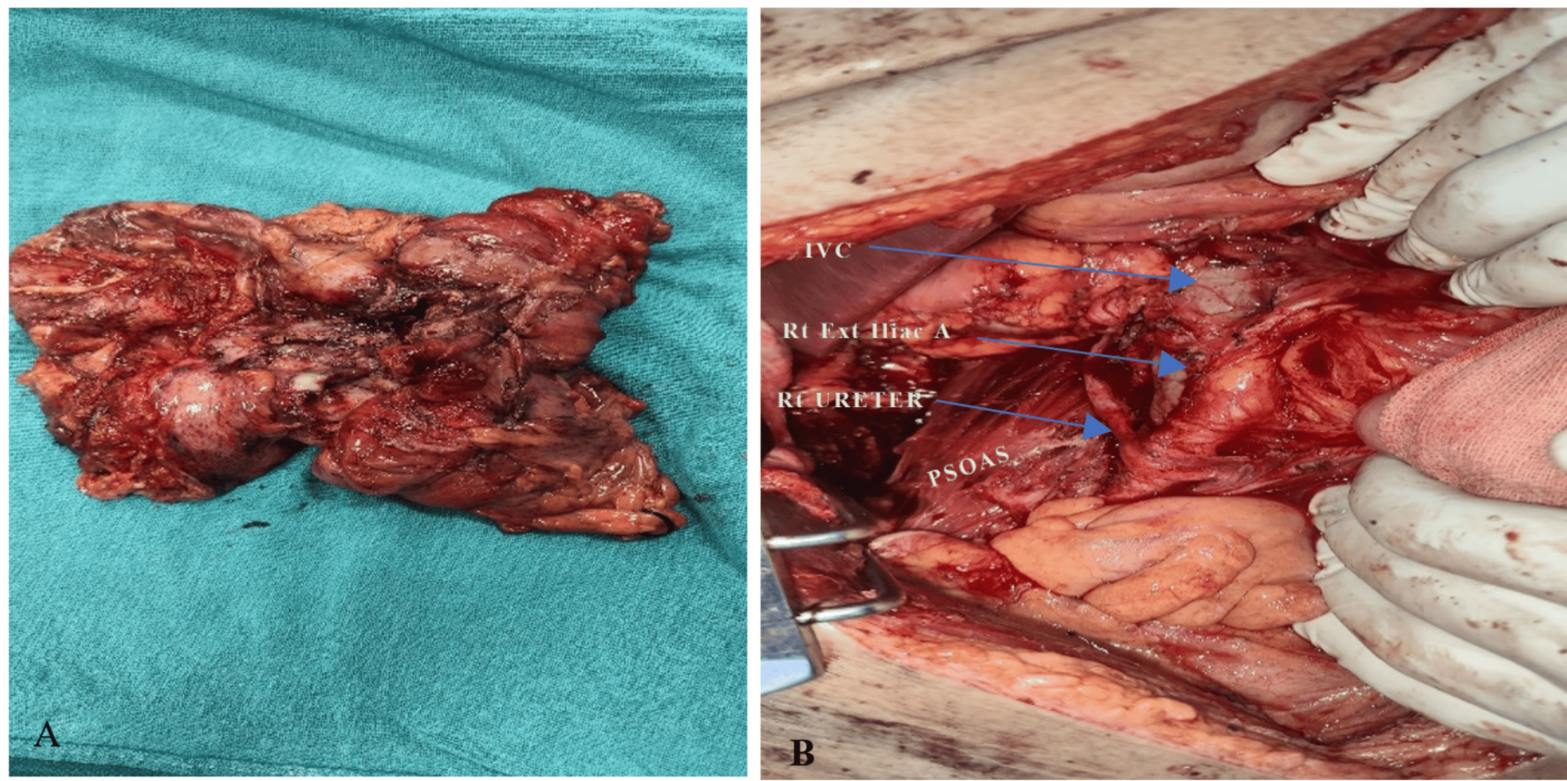
Gross and Intraoperative Findings A) Resected specimen of the retroperitoneal cystic mass along with the resected right colon. B) Intraoperative picture of tumor bed, showing displaced right kidney, psoas muscle, and critical structures marked with arrows.

Based on clinical, laboratory, and imaging findings, a provisional diagnosis of retroperitoneal cystic neoplasm was made, and the patient was planned for exploration. Intraoperatively, a large retrocolic retroperitoneal cystic mass was identified, which was densely adherent to the ascending and hepatic flexure colon and psoas muscle. Abutting and displacing the right kidney, right ureter, right external iliac artery, and parietal abdominal wall (Figure 2). The liver and the right kidney were free from the cyst. Dense adhesions were encountered with the ascending colon and in the paravertebral area. Complete retroperitoneal cystic mass excision with right-sided hemicolectomy with end-end ileo-colic anastomosis was done.

The patient's postoperative course was uneventful. He was allowed orally on postoperative day (POD)-2, the pelvic drain was removed on POD-5, and he was discharged on POD-10.

On gross section, the tumor measured 14 × 15 × 10 cm. The tumor was primarily cystic, filled with gelatinous material, with a few solid components within. Histopathology was suggestive of moderately differentiated mucinous adenocarcinoma. Tumor cells invade up to the pericolic fat. Resected margins were of the tumor without any perineural or lymphovascular invasion. Three lymph nodes (0/3) were identified, all of which showed non-specific inflammation.

**Figure 3:**
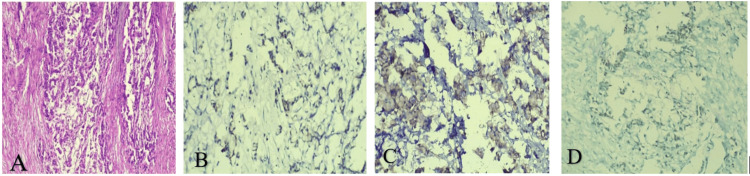
Microscopy and Immunohistochemistry (IHC) Images A) Magnified view of the lesion on hematoxylin and eosin staining (×100) shows large atypical cells with chromatin-rich nuclei arranged singly or in small clusters. Cystic areas are lined by mucinous epithelium displaying borderline papillary features. B) Nuclear staining with CDX 2 marker. C) IHC slide showing positive cytoplasmic staining in glandular epithelial cells; CK20 is normally expressed in gastrointestinal epithelium. D) Positive nuclear staining in tumor cells with the SATB2 marker.

Immunohistochemistry (IHC) for further characterization showed the tumor was positive for CDX2, CK20, and SATB2 (Figure 3). The above finding strongly suggested that the tumor has intestinal-type differentiation, most consistent with a colorectal-type mucinous adenocarcinoma phenotype, even though it is arising in the retroperitoneum. Based on these histopathological findings, a diagnosis of primary retroperitoneal mucinous adenocarcinoma (pT4aN0Mx) was established. The oncology team followed up with the patient and subsequently started on adjuvant chemotherapy (paclitaxel + carboplatin-based regimen).

## Discussion

PRMC is a rare malignancy arising from the retroperitoneum, commonly featuring mucinous cystic components. The rarity of this lesion in the retroperitoneal region is attributed to the lack of native epithelial cells in this area [4,5]. The etiology and biological behavior of PRMC remain unclear. The majority of PRMCs are found in females. The first case in a male patient was reported by Motoyama et al. in 1994 [1], with limited details regarding clinical presentation and follow-up. Although the exact etiology remains unclear, proposed theories suggest that the tumor may arise from ectopic ovarian tissue, accounting for its predominance in females, or from mucinous metaplasia. The latter involves multipotent mesothelial cells in the retroperitoneum undergoing mucinous metaplastic transformation, leading to the development of such lesions. Other theories suggest that it may occur as a monodermal variant of teratomas or embryonal urogenital remnants [6].

PRMC often presents with vague symptoms, making early diagnosis difficult due to the anatomical characteristics of the retroperitoneum. The clinical course is generally slow and indolent; however, these tumors have the potential to exhibit aggressive behavior. A CT scan is preferred for assessing anatomical details, detecting calcifications, and identifying fat components owing to its high spatial resolution. MRI, on the other hand, offers superior soft tissue contrast and is more effective in evaluating vascular involvement.

Histopathological evaluation is crucial for diagnosis, as these tumors are clinically and radiologically challenging to distinguish and are often misidentified as renal masses. Histologically, mucinous neoplasms are categorized into three types: benign, borderline, and malignant [7,8].

Benign mucinous cystadenoma is the most frequent subtype and carries an excellent prognosis. Borderline mucinous cystadenomas, characterized by papillary projections, are less common. Malignant tumors are rare, demonstrate invasive features, and are associated with a poor prognosis. Accurate preoperative diagnosis is often difficult; therefore, complete surgical excision with clear margins remains the mainstay of treatment. Adjuvant therapy may be considered in cases exhibiting unfavorable prognostic features or recurrence. Radiologically, cyst wall thickening, calcifications, or mural nodules may raise suspicion for malignancy. Aspiration cytology of cyst fluid can assist in diagnosis, and CEA and CA19-9 levels may occasionally be elevated in serum or cyst fluid samples. When adenocarcinoma is identified on biopsy, immunohistochemical evaluation is essential to rule out metastasis from other primary sites, as adenocarcinomas rarely originate in the retroperitoneum. PRMC typically shows CK7 positivity and CK20 negativity. In our case, the tumor was positive for CDX2, CK20, and SATB2 (Figure 3).

Adjuvant chemotherapy is advised in cases with tumors (>10 cm in size), high histologic grade, mucinous type, lymphovascular invasion, perineural invasion, positive margins status, lymph node involvement, intraoperative spillage, and persistently high postoperative tumor marker levels. The presence of solid areas, mural nodules, and sarcoma-like or anaplastic components has been found to be associated with the aggressive behavior of this tumor [9]. PRMC in males is exceptionally rare, with only about 10 cases reported in the literature to date [10]. Males appear to have a higher risk of developing malignant retroperitoneal lesions, as all reported cases in men have shown a malignant focus. Postoperative follow-up using serum markers, if elevated before surgery, can aid in monitoring for recurrence. Due to the high risk of relapse, lifelong follow-up is recommended. Five-year survival is up to 70-80% for completely resected, low-grade tumors and <30% for high-grade or recurrent disease.

## Conclusions

To conclude, PMRC is an extremely rare neoplasm in males, with only a handful of cases reported in the literature. Complete surgical excision with negative margins remains the mainstay of treatment, with adjuvant chemotherapy. IHC findings suggestive of intestinal type differentiation and aggressive biology (mucinous) warrant lifelong surveillance due to the risk of recurrence. Reporting such rare cases contributes to a better understanding of the biological behavior, diagnostic dilemmas, and management strategies of this unusual tumor.
